# Enhancing CO_2_ Fixation in Microalgal Systems: Mechanistic Insights and Bioreactor Strategies

**DOI:** 10.3390/md23030113

**Published:** 2025-03-07

**Authors:** Zhongliang Sun, Chenmei Bo, Shuonan Cao, Liqin Sun

**Affiliations:** College of Life Sciences, Yantai University, Yantai 264005, China

**Keywords:** microalgae, photobioreactor systems, CO_2_ supplementation techniques, large-scale deployment

## Abstract

Microalgae are small, single-celled, or simple multicellular organisms that contain Chlorophyll a, allowing them to efficiently convert CO_2_ and water into organic matter through photosynthesis. They are valuable in producing a range of products such as biofuels, food, pharmaceuticals, and cosmetics, making them economically and environmentally significant. Currently, CO_2_ is delivered to microalgae cultivation systems mainly through aeration with CO_2_-enriched gases. However, this method demonstrates limited CO_2_ absorption efficiency (13–20%), which reduces carbon utilization effectiveness and significantly increases carbon-source expenditure. To overcome these challenges, innovative CO_2_ supplementation technologies have been introduced, raising CO_2_ utilization rates to over 50%, accelerating microalgae growth, and reducing cultivation costs. This review first categorizes CO_2_ supplementation technologies used in photobioreactor systems, focusing on different mechanisms for enhancing CO_2_ mass transfer. It then evaluates the effectiveness of these technologies and explores their potential for scaling up. Among these strategies, membrane-based CO_2_ delivery systems and the incorporation of CO_2_ absorption enhancers have shown the highest efficiency in boosting CO_2_ mass transfer and microalgae productivity. Future efforts should focus on integrating these methods into large-scale photobioreactor systems to optimize cost-effective, sustainable production.

## 1. Introduction

Microalgae have the capability to fix CO_2_ through photosynthesis, producing a range of valuable chemicals. Certain species, such as *Botryococcus braunii*, can generate hydrocarbons that constitute 15% to 75% of their dry weight. Other species accumulate glycogen or glycerol, and many have lipid contents exceeding 30% of their dry weight [1,2]. The pyrolysis of microalgal biomass can produce biofuels with an average calorific value of up to 33 MJ/kg [3]. Moreover, microalgae can be cultivated in seawater, alkaline water, or semi-alkaline water, which helps avoid competition with crops for arable land and freshwater. They can also utilize nitrogen-rich wastewater, making them a valuable resource in areas with limited freshwater and degraded land [4,5]. Thus, microalgae present a promising future source of energy and chemicals.

The carbon content in microalgal cells represents about half of their dry weight. During growth, microalgae fix CO_2_ into their cellular components through photosynthesis, necessitating a continuous supply of carbon sources in the cultivation medium [6]. In the medium, inorganic carbon exists in three forms: HCO_3_^−^, CO_3_^2^^−^, and free CO_2_. The concentration and ratio of these forms depend on the total inorganic carbon concentration and pH [7]. For most economically valuable microalgal species, the optimal growth pH ranges from 6 to 8, during which the primary forms of inorganic carbon in the medium are free CO_2_ and HCO_3_^−^. In contrast, alkaliphilic species, such as *Spirulina*, thrive at a pH of around 9.0, where the dominant carbon species are HCO_3_^−^ and CO_3_^2^^−^. When sodium bicarbonate (NaHCO_3_) is used, the medium’s pH increases due to the dissociation of HCO_3_^−^ and CO_2_ consumption. This process can convert more than half of the NaHCO_3_ into Na_2_CO_3_, which is not usable by the microalgae and complicates medium recycling due to the elevated pH [8]. Using CO_2_ directly as a carbon source is more effective for microalgae, as it avoids the issue of rising pH and helps maintain an optimal cultivation environment, allowing for extended or repeated use of the medium.

Microalgal cultivation methods are generally divided into open and closed systems [9]. Both typically involve bubbling CO_2_-enriched gas into the cultivation medium, but this method is inefficient due to low CO_2_ absorption rates (13–20%), leading to high carbon-source costs [10,11]. Since carbon accounts for approximately half of the dry weight of microalgal cells, an increase of 1 g/L in cell concentration requires the assimilation of around 2 g/L of CO_2_. However, the solubility of pure CO_2_ in water is relatively low, at only 1.45 g/L at 25 °C, and even lower when CO_2_ is sourced from air, with a solubility of just 0.58 mg/L under the same conditions. This limited solubility poses a significant challenge for maintaining sufficient inorganic carbon availability in the cultivation medium. Therefore, enhancing the concentration of inorganic carbon species and improving CO_2_ absorption efficiency are critical for supporting rapid microalgal growth and minimizing cultivation costs.

Recent advancements have led to the development of innovative CO_2_ supplementation technologies designed to meet the rapid growth requirements of microalgae while reducing cultivation costs. Examples include in situ CO_2_ supplementation devices in raceway ponds, which increase gas–liquid contact time and surface area, and methods that improve CO_2_ absorption and conversion using immobilized carbonic anhydrase [7,12,13]. This review categorizes these CO_2_ supplementation technologies based on their mechanisms for enhancing CO_2_ mass transfer, assesses their effectiveness, and explores the potential for scaling up these technologies. By systematically addressing these objectives, this review aims to offer a comprehensive understanding of CO_2_ management in microalgal cultivation and highlight innovative strategies to overcome the limitations of conventional carbon-supplementation methods.

## 2. Methodologies and Devices for Enhancing CO_2_ Mass Transfer in Microalgal Systems

### 2.1. CO_2_ Mass-Transfer Process

Under photoautotrophic growth conditions, microalgae use inorganic carbon sources to synthesize organic compounds and convert light energy into chemical energy. Microalgae can absorb both CO_2_ and HCO_3_^−^ but cannot utilize CO_3_^2^^−^ [14]. CO_2_ enters the cells through diffusion and is used directly, while HCO_3_^−^, being a polar and negatively charged ion, requires active transport across the cell membrane, a process that consumes energy [15]. Consequently, the absorption of HCO_3_^−^ is slower compared to CO_2_, though some algae species that thrive in high pH environments, such as *Spirulina*, exhibit better HCO_3_^−^ absorption.

During CO_2_ transfer in the cultivation medium, it can react with OH^−^ and CO_3_^2^^−^, though these reactions have minimal impact on CO_2_ transfer efficiency [16]. In microalgal culture media, CO_2_ transfer is a multi-step process involving several stages: from the gas phase to the gas film, diffusion within the gas film, transfer from the gas film to the liquid film, diffusion through the liquid film, movement from the liquid film into the liquid phase, diffusion within the liquid phase, transfer from the liquid phase to the liquid film at the cell-wall surface, and finally, cellular absorption. According to the two-membrane theory, the primary resistance to CO_2_ transfer occurs within the liquid film, which serves as the main barrier limiting the efficiency of gas–liquid mass transfer [17,18]. The mass-transfer rate is proportional to the driving force and the area available for mass transfer. The rate of CO_2_ transfer can be expressed as:

(1) $$N_{CO_{2}}=K_{L,a}(C_{CO_{2},L}^{*}-C_{CO_{2},L})$$

where *K_L,a_* is the overall liquid volumetric mass-transfer coefficient for the absorption of CO_2_, dependent on factors like phase contact area and the intensity of gas–liquid mixing. $C_{CO_{2},L}^{*}$ represents the CO_2_ concentration in the liquid phase at equilibrium with the gas-phase concentration.

### 2.2. In Situ CO_2_ Supplementation

**Figure 1:**
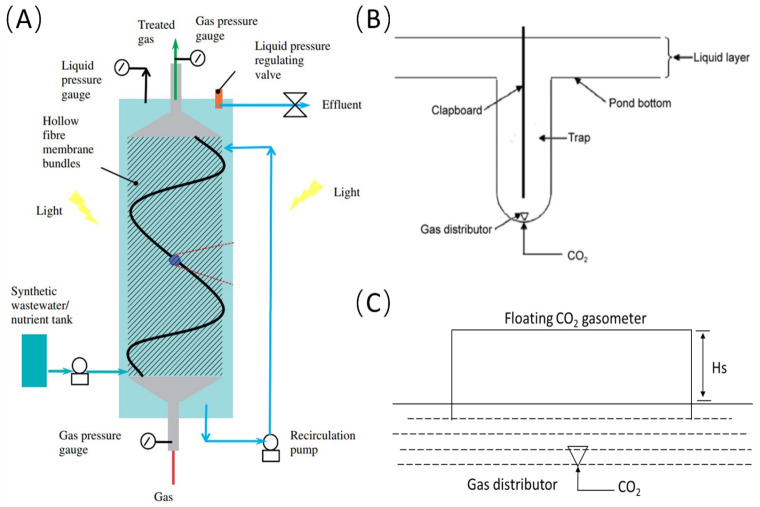
In situ carbon-supplementation devices in microalgae cultivation systems; (**A**) hollow fiber membrane module; (**B**) trap-type carbon-supplementation device; (**C**) leak-proof cover device.

To enhance CO_2_ absorption in algal medium, several in situ CO_2_ supplementation devices have been developed. Kumar et al. improved CO_2_ mass transfer by using hollow-fiber membranes, which provide a significantly larger interphase contact area compared to traditional bubbling methods, resulting in a mass-transfer coefficient approximately ten times greater (Figure 1A) [19]. This technology enhances CO_2_ absorption efficiency and facilitates CO_2_ recycling, thereby reducing cultivation costs [20]. Ketheesan et al. introduced a novel airlift raceway-pond design where CO_2_ is injected into an ascending channel, increasing CO_2_ and liquid contact time and achieving a 50% absorption rate [21]. Our research team implemented an in situ CO_2_ supplementation trap device in an open raceway pond for *Spirulina platensis* cultivation (Figure 1B) [22]. This device, featuring a trap container, partition, and gas distributor, effectively extended the gas–liquid contact time from 3 s to 8 s and enhanced CO_2_ utilization efficiency to over 90% (Table 1). Chen et al. utilized a leak-proof cover over the cultivation layer, which collected CO_2_ and created a large gas–liquid exchange area (Figure 1C), though challenges included limited gas–liquid exchange surface area, the accumulation of oxygen and nitrogen, and reduced light transmittance [23]. Our research team also developed a submerged cover-type CO_2_ supplementation device installed at the bottom of open ponds. Transparent glass covers above the aeration points and below the liquid surface allowed bubbles to have extended contact time with the liquid, reducing CO_2_ escape and improving CO_2_ absorption efficiency [24].

**Figure 2:**
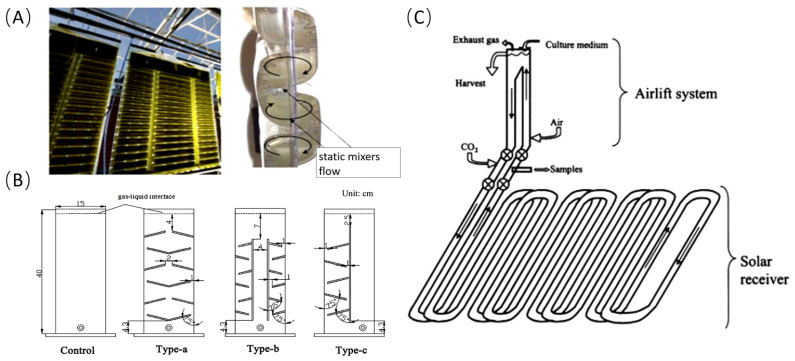
Closed photobioreactor and structural modifications for enhanced CO_2_ mass transfer; (**A**) multi-chamber structure of flexible flat-panel reactor; (**B**) different types of baffle structures in flat-panel reactors; (**C**) tubular reactor and CO_2_ supplementation positions.

In closed photobioreactors, Bergmann et al. enhanced bubble residence time by modifying flat-panel reactors to multiple chambers, achieving over 80% CO_2_ absorption (Figure 2A) [25]. Huang et al. further enhanced gas–liquid mixing in flat-panel reactors by incorporating disturbance columns or inclined baffles (Figure 2B). This modification increased mixing intensity by up to 52%, significantly boosting the CO_2_ mass-transfer coefficient [26]. The tubular photobioreactor, currently the most widely used closed photobioreactor, has evolved through multiple generations into a structure comprising light absorption units, gas–liquid exchange units, and circulation pumps. CO_2_ can be introduced into the gas–liquid exchange unit or before the culture liquid enters the light absorption unit, or at a specific position within the light absorption unit. Under the action of the circulation pump, the gas moves with the liquid and is gradually absorbed, resulting in high CO_2_ absorption rates (Figure 2C) [27].

**Table 1:** Effect of methodologies and devices on microalgae growth and CO_2_ utilization efficiency.

Microalgal Culture System	Methodologies or Devices	Species	Biomass	CO_2_ Utilization Efficiency	Mechanisms	Reference
Photobioreactor	Hollow fiber membrane	*Spirulina platensis*	2131 mg/ L ↑	85% ↑	Increase the interfacial contact area available for gas transfer	[19,20]
Raceway pond	An ascending channel	*Scenedesmus* sp.	0.16 ± 0.03 g/(L·d) ↑	50% ↑	Increase mixing intensity	[21]
Raceway pond	CO_2_ supplementation trap device	*Spirulina platensis*	3.45–6.04 g/(m^2^·d) ↑	90% ↑	Prolong gas–liquid contact time	[22]
Open pond	Leak-proof cover	*Cyanobacterium* sp.	2.5 g/L ↑	80% ↑	Create a large gas–liquid exchange area	[23]
Open pond	Submerged cover-type	*Spirulina platensis*	13.3 g/(m^2^·d) ↑	92% ↑	Prolong gas–liquid contact time	[24]
Photobioreactor	Multiple chambers	*Nannochloropsis salina*	0.12 g/(L·d) ↑	80% ↑	Enhance bubble residence time	[25]
Flat-plate PBRs	Inclined baffles	*Chlorella pyrenoidosa*.	1.3 g/ L	No data	Increase mixing intensity	[26]
Raceway pond	Vertical absorption tower	*Chlorella pyrenoidosa*.	20 g/(m^2^·d) ↑	83% ↑	Prolong gas–liquid contact time	[28]
Open pond	Absorption tank	*Spirulina platensis*	6–12 g/(m^2^·d)	>50%	Increase mixing intensity	[4,29]

↑ indicates that the indicators of the experimental group have improved compared with the control group.

### 2.3. Ex Situ CO_2_ Supplementation

**Figure 3:**
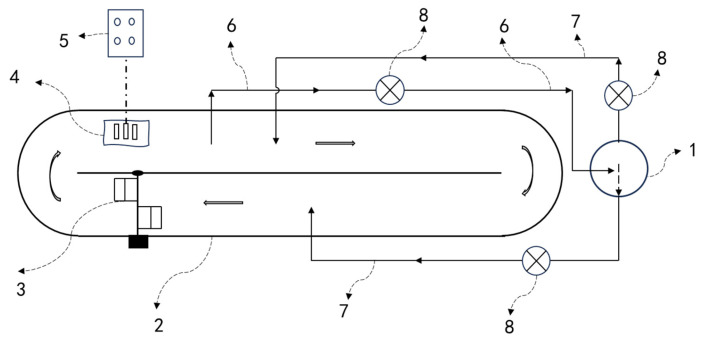
Microalgae cultivation system equipped with ex situ CO_2_ supplementation devices. (1) vertical column CO_2_ absorption tower, or CO_2_ absorption tank, (2) open raceway pond, (3) paddle wheel, (4) pH/O_2_ electrode, (5) control system, (6) medium outlet pipe, (7) medium inlet pipe, (8) circulation pump.

In addition to in situ carbon-supplementation devices in photobioreactor systems, some ex situ carbon-supplementation devices have also been developed. Putt et al. set up a vertical absorption tower outside the raceway pond, with a dynamic pump driving the culture medium to circulate between the vertical absorption tower and the raceway pond, achieving a CO_2_ absorption rate of 83% [28]. Trench-type carbon supplementation involves excavating a deep trench adjacent to the cultivation pond, allowing the culture medium to flow through it, with aeration pipes installed at the trench bottom to supply CO_2_ [30]. In practical applications, these carbon-supplementation trenches are often designed in a funnel or conical shape to enhance flow dynamics. However, this method disrupts the conventional spatial configuration of open ponds, and over time, CO_2_ can accumulate at the trench bottom, creating a mass-transfer dead zone and reducing the system’s overall effectiveness. Increasing the aeration rate can mitigate the formation of dead zones, but it inevitably shortens bubble residence time, leading to greater CO_2_ escape into the atmosphere. During large-scale *Spirulina* cultivation, the culture liquid, after being enriched with CO_2_ in a carbon-supplementation tank, is returned to the cultivation pond for photosynthetic production [4,29]. The development and application of these carbon-supplementation technologies have increased the annual production of *Spirulina* by 20%, reduced annual sodium bicarbonate usage by 66%, and lowered carbon-source costs by 58% [31] (Figure 3).

In summary, CO_2_ supplementation strategies that extend gas–liquid contact time or enhance gas–liquid mixing intensity often come at the cost of increased energy consumption. For instance, the incorporation of CO_2_ supplementation trenches in raceway ponds prolongs bubble residence time; however, maintaining the same liquid flow rate results in an 80% increase in the energy consumption of the paddlewheel system [21]. In contrast, membrane-based technologies enhance CO_2_ mass-transfer efficiency without significantly increasing energy demand. This approach diffuses CO_2_ into the liquid medium through a nonporous hollow-fiber membrane, eliminating macroscopic bubbles. It achieves three times the CO_2_ mass-transfer efficiency of conventional sparging while avoiding the shear forces associated with micro- and nano-bubbles that can damage microalgal cells.

## 3. Strategies for Enhancing CO_2_ Mass Transfer Using Chemical Solvents

According to the two-membrane theory and the CO_2_ mass-transfer model in the culture medium (Equation (1)), the primary resistance to CO_2_ transfer occurs at the liquid membrane interface. In addition to increasing the overall volumetric mass-transfer coefficient by enhancing gas–liquid mixing intensity, increasing the gas–liquid contact specific area, and extending the gas–liquid contact time, the efficiency of CO_2_ mass transfer in the culture medium can also be improved by introducing chemical reactions and altering the physical properties of the culture medium [32,33].

### 3.1. Novel Enhancing Mechanism of Introducing Chemical Reaction

During CO_2_ transfer in the culture medium, it reacts with OH^−^ and CO_3_^2−^ present in the medium. However, it has been indicated that these chemical reactions have minimal impact on CO_2_ transfer and absorption, primarily because the optimal pH for most microalgae is close to neutral, resulting in low concentrations of OH^−^ and CO_3_^2−^ in the medium under such conditions. If a high-concentration alkaline solution is thoroughly contacted with CO_2_-containing gas in an ex situ absorption tower (Figure 3), the resulting carbon-rich solution can be used as a carbon source for microalgae cultivation, ensuring high CO_2_ absorption rates and meeting the substantial carbon-source demand of microalgae. Zhu et al. used NaOH and Na_2_CO_3_ solutions as absorbents, and after convective mass transfer with CO_2_-containing gas, the primary inorganic carbon species in the resulting CO_2_-enriched solution was HCO_3_^−^. This solution was used as a carbon source to achieve high-density cultivation of *Spirulina*, alkali-resistant *Oscillatoria*, and *Isochrysis galbana*, resulting in high CO_2_ utilization rates in closed floating photobioreactors [34,35,36]. However, as previously mentioned, the continuous proliferation of microalgal cells leads to an increase in the pH of the culture medium, promoting the conversion of HCO_3_^−^ into CO_3_^2^^−^. Since CO_3_^2^^−^ is not a bioavailable form of inorganic carbon for microalgae, this results in carbon-source wastage. Moreover, the use of NaOH or Na_2_CO_3_ solutions as absorbents elevates the Na^+^ ion concentration in the culture medium, increasing extracellular osmotic pressure and adversely affecting microalgal growth. For instance, *Chlorella*, a typical freshwater microalga, can tolerate salinity levels of only 5–10‰. In addition to inhibiting growth, elevated salinity complicates the recycling and reuse of the culture medium, further reducing the efficiency of the cultivation process [37,38].

Our research team has explored the strategy of using “ammonium hydroxide” as a nitrogen source to enhance microalgae growth and CO_2_ absorption. The primary product of CO_2_ absorption by ammonia water is ammonium bicarbonate, which can be directly used by microalgae cells as both a carbon and nitrogen source without introducing additional metal ions. This strategy can significantly reduce nutrient costs in microalgae cultivation [39]. By combining pH-feedback CO_2_ supplementation strategies with the metabolic kinetics of microalgae cells regarding nitrogen sources, stable control of low-concentration ammonium salts in the culture medium can be achieved, avoiding the inhibition of algal-cell growth and nitrogen-source loss due to ammonia volatilization [40]. Furthermore, using a closed gas-lift reactor for cultivating *Chlorella* sp., CO_2_ utilization rates reached 87.8%. In open raceway ponds, using a simple bottom-bubbling method for CO_2_ supplementation resulted in a CO_2_ utilization rate of 35.58%, which is an increase of 14.46% compared to the control group. However, 1 mol of ammonia water absorbs approximately 0.3–0.8 mol of CO_2_. Based on the elemental composition of microalgae cells (CH_1.911_O_0.496_N_0.196_P_0.007_S_0.005_), the required C/N ratio for the culture medium is approximately 5 [41,42]. Thus, when using the “ammonium hydroxide” strategy for microalgae cultivation, a substantial amount of CO_2_ must still be supplemented to meet the fast growth requirements for carbon sources.

Amines are commonly used CO_2_ absorbents in carbon capture, storage, and utilization (CCUS) applications [43]. These include monoethanolamine (MEA), diethanolamine (DEA), triethanolamine (TEA), and N-methyldiethanolamine (MDEA), which react reversibly with CO_2_ as follows:

(2) $$CO_{2}+RNH_{2}⇄RNHCOO^{-}+H^{+}$$

(3) $$RNHCOO^{-}+H_{2}O⇄RNH_{2}+HCO_{3}^{-}$$

The impact of adding amine-based CO_2_ absorbents to microalgae cultivation systems on carbon-source utilization and microalgae growth have been investigated by our team and the other researchers (Table 2) [44,45,46,47,48,49,50,51,52,53,54,55,56,57,58,59,60,61,62,63,64,65,66,67]. The introduction of chemical reactions increases the CO_2_ mass-transfer rate (with a chemical absorption enhancement factor β > 1.0) [32]. Additionally, the resulting carbamate (RNHCOO^−^) acts as a “CO_2_ carrier”, becoming the fourth form of carbon species in the culture medium. Under near-neutral pH conditions, as microalgae cells consume CO_2_ and HCO_3_^−^, HCO_3_^−^ is gradually released from the carbamate, acting as a slow-release carbon reservoir. It is indicated that the addition of amine-based CO_2_ absorbents can enhance the biomass productivity of *Spirulina*, and *Chlorella*. In column reactors, CO_2_ utilization efficiency increased from 44.5% to 76.1% [46,47,48,49,50]. It is worth noting that most amine molecules are not easily metabolized by microalgae cells, allowing them to act as a repeated CO_2_ capture agent in the culture medium [48]. However, Rosa et al. found that high concentrations of chemical absorbents can inhibit microalgae growth. For instance, when MEA concentrations exceed 150 mg/L, microalgae biomass productivity decreases, and cell growth and intracellular metabolic activity are suppressed [51,52]. This phenomenon may be attributed to the corrosive nature of high-concentration amine solutions. Therefore, selecting an appropriate “CO_2_ carrier” requires careful consideration of its biocompatibility.

### 3.2. Novel Enhancing Mechanism of Altering the Medium’s Physical Properties

In industrial applications, methods for CO_2_ capture from flue gases also include low-temperature methanol methods (Restisol process), ethylene glycol ether methods, propylene carbonate methods (Flour process), and N-methyl-2-pyrrolidone methods [53,54]. These compounds do not chemically react with CO_2_ but enhance CO_2_ solubility in the liquid phase by altering the physicochemical properties of the absorbent, such as reducing surface tension. Since CO_2_ solubility in solvents follows Henry’s Law, these absorbents generally have lower Henry’s coefficients compared to aqueous solvents. Therefore, adding such absorbents to microalgae cultivation systems effectively increases the equilibrium concentration of CO_2_ in the liquid phase, thereby promoting CO_2_ absorption according to the mass-transfer model (Equation (1)) [50,55,56].

Using gas-lift photobioreactors to cultivate *Chlorella.* sp., our research indicated that the addition of four types of absorption enhancers—methanol, NHD, PC, and NMP—can significantly increase CO_2_ utilization rates during cultivation, with an optimal condition improving CO_2_ utilization by 71%, without significantly affecting the biochemical composition of the microalgae [55]. A method is utilized where a water-immiscible solvent is directly added to a microalgal culture, simultaneously allowing for increased CO_2_ absorption by the algae while also extracting lipids from the cells in a single process, eliminating the need for separate cultivation and extraction steps; often, n-heptane is used as the water-immiscible solvent due to its ability to act as a “CO_2_ sink” and readily extract lipids without significantly harming the algal cells [50].

## 4. Carbonic Anhydrase-Assisted CO_2_ Absorption and Conversion

**Figure 4:**
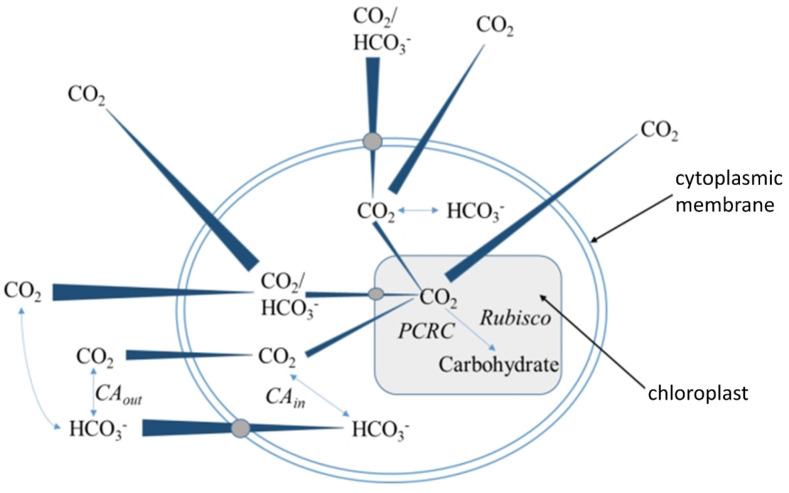
Diagrammatic representation of CO_2_ absorption and conversion pathways in microalgae cells.

CO_2_ absorption and conversion in microalgae culture can be broadly divided into two stages: gas–liquid mass transfer and biological conversion (Figure 4). After the gas–liquid mass transfer, the dissolved inorganic carbon sources include CO_2_ and HCO_3_^−^. The latter is further absorbed by algal cells through two main pathways: active transport via membrane carrier proteins or conversion into CO_2_ molecules under the action of extracellular carbonic anhydrase, which then rapidly diffuses into the cell [57,58,59]. Thus, highly active extracellular carbonic anhydrase facilitates CO_2_ biological conversion, reducing the concentration of inorganic carbon sources in the liquid phase and promoting CO_2_ gas–liquid mass transfer (Table 2). However, most microalgae exhibit low carbonic anhydrase activity under near-neutral conditions [60,61]. The addition of exogenous carbonic anhydrase may enhance microalgae’s ability to absorb and convert CO_2_, thereby influencing cell growth rates. However, the stability and durability of carbonic anhydrase in photobioreactors are currently poor [62]. In bubbling gas–liquid environments, shear forces significantly degrade enzyme activity, increasing the cost of microalgae cultivation beyond nutrient input and limiting the application of this strategy in microalgae cultivation technologies. The setup of efficient methods for enzyme immobilization makes carbonic anhydrase utilization in continuous bioreactors increasingly attractive and opens up new opportunities for the industrial use of carbonic anhydrase [12,63].

Xu et al. proposed a technique involving the addition of immobilized carbonic anhydrase microbeads to microalgae culture, which effectively addressed the issue of carbon limitation when cultivating microalgae with air CO_2_ as the sole carbon source [13]. Jun et al. fixed carbonic anhydrase onto electrospun polymer nanofibers using enzyme deposition coating. This approach increased the microalgae growth rate by 134% in a bubbling reactor [64]. Yang et al. developed carbonic anhydrase-coated nylon fiber membranes and proposed a novel photobioreactor that improves CO_2_ solubility and absorption rates in the microalgae solution. Testing revealed that CO_2_ conversion rates increased by 62.7% with the use of this immobilized exogenous carbonic anhydrase [65]. Our team has isolated mineralizing bacteria from soil environments that symbiotically coexist with *Chlorella* sp. These symbiotic bacteria can extensively express extracellular carbonic anhydrase. By optimizing algal–bacterial inoculation ratios and other parameters, we have developed a microalgae cultivation system that enhances CO_2_ absorption through immobilized mineralizing bacteria [66]. Wang et al. utilized bamboo cellulose as a renewable porous scaffold to immobilize carbonic anhydrase through oxidation-induced aldehyde formation, followed by Schiff base linkage. The cellulose-immobilized enzyme significantly enhanced microalgal growth and biomass accumulation [67].

## 5. Challenges and Future Prospects

In the large-scale cultivation of alkaliphilic microalgae such as *Spirulina* (with an optimal pH range of 9.0–11.0), the combined use of CO_2_ supplementation devices and pH-feedback CO_2_ supplementation strategies can achieve carbon-source utilization rates exceeding 80%, effectively reducing cultivation costs. This is primarily due to higher CO_2_ absorption rates under alkaline conditions and the higher total inorganic carbon concentration in the culture medium (maintained by adding large amounts of NaHCO_3_), which supports rapid microalgal growth and carbon-source demands. In near-neutral (pH 6.0–8.0) cultivation systems (e.g., *Chlorella*, *Scenedesmus*, *Microcystis*, *Euglena*, and other economically significant algae), it is necessary to control the total inorganic carbon concentration in the carbon-rich culture medium from the supplementation device to prevent the loss of free CO_2_ during circulation. This results in a lower total inorganic carbon concentration that only supports short-distance flow, depleting the carbon source and affecting algal biomass productivity.

For example, in a typical microalgae cultivation process with pH = 7, a liquid layer depth of 20 cm, an area productivity of 15 g/(m^2^·d), and 30 °C, if the CO_2_ in the culture medium is in equilibrium with the air upon leaving the supplementation point (meaning no CO_2_ loss to the air), calculations show that the total inorganic carbon concentration drops to zero after 2 min of flow (although, in practice, microalgae growth is limited before reaching zero). At a flow velocity of 20 cm/s, this equates to a distance of 24 m. Thus, in fixed-scale raceway ponds, such as those with a perimeter of over 200 m in large-scale production, multiple carbon-supplementation points are needed to reduce CO_2_ loss, ensure high CO_2_ utilization rates, and maintain high algal-cell productivity. For a raceway pond with a single carbon-supplementation point and a perimeter of 200 m, the low concentration of available carbon sources quickly leads to carbon-source limitation after leaving the supplementation point, impacting algal biomass productivity. Increasing the carbon-source concentration at the supplementation point forces CO_2_ to escape, exacerbating losses.

Thus, CO_2_ supplementation in microalgal photobioreactor systems must achieve both high CO_2_ absorption rates at supplementation points and continuously increase the total inorganic carbon concentration in the culture medium. Future research should focus on multi-scale studies and the precise application of various strategies to optimize the CO_2_ absorption process for microalgae.

Firstly, the kinetics of microalgae CO_2_ absorption and conversion should be comprehensively studied. Utilize visual experimental methods to explore the kinetics of CO_2_ dissolution and mass transfer, identify rate-limiting steps, and establish a balance between CO_2_ supplementation, dissolution, transfer, and conversion processes. Precise control strategies and models should be developed. Secondly, create new and efficient microalgae cultivation systems by integrating in situ or ex situ setups with photobioreactor systems. Investigate low-energy methods for bubble nanonization, combine gas–liquid mixing with membrane technologies, and improve CO_2_ mass-transfer efficiency under near-neutral pH conditions. Finally, select compounds or enzyme preparations with good biocompatibility that increase CO_2_ solubility through chemical reactions or alterations in the physicochemical properties of the culture medium. This will enhance the CO_2_ sink in microalgae cultivation systems, meet the rapid carbon-source demands of microalgae growth, reduce the frequency of carbon supplementation, and save energy.

## 6. Conclusions

This review highlights the various CO_2_ supplementation strategies aimed at enhancing the efficiency of microalgae cultivation for biotechnological applications. Traditional aeration methods often result in low CO_2_ absorption rates, which hinder microalgal growth and biomass productivity. To address these limitations, several innovative approaches have been developed. Direct CO_2_ injection and advanced open-pond designs, such as trap-type carbon-replenishing devices and membrane-based systems, significantly enhance CO_2_ mass-transfer efficiency. Modifications to closed photobioreactors further improve CO_2_ utilization. Additionally, CO_2_ absorption enhancer or immobilized carbonic anhydrase plays a crucial role in optimizing CO_2_ absorption and promoting algal growth. Among these strategies, membrane-based CO_2_ delivery systems and the incorporation of CO_2_ absorption enhancers have shown the highest efficiency in boosting CO_2_ mass transfer and microalgae productivity. Future efforts should focus on integrating these methods into large-scale photobioreactor systems to optimize cost-effective, sustainable production.

## Figures and Tables

**Table 2:** Effect of adding chemical absorbents or immobilized enzymes on microalgae growth and CO_2_ utilization efficiency.

Absorbents or Immobilized Enzymes	CO_2_ Content	Species	Biomass	CO_2_ Utilization Efficiency	Reference
100 mg/L MEA	10%	*Scenedesmus dimorphus*	0.293 g/(L·d) ↑	76.1% ↑	[44]
6 mmol/L THAM	100%	*Scenedesmus dimorphus*	11.57 g/(m^2^·d) ↑	35.58% ↑	[45]
2 mmol/L TEA	4%	*Scenedesmus* sp.	0.664 g/(L·d) ↑	No data	[46]
1.64 mol/L EDA + 0.41 mmol/L K_2_CO_3_	0.04%	*Spirulina* sp. LEB18	0.174 g/(L·d) ↑	No data	[47]
12% N-heptane	15%	*Chlorella* sp.	0.084 g/(L·d) ↑	64.7% ↑	[48]
100–150 mg/L MEA	50%	*Chlorella fusca LEB 111*	0.096–0.122 g/(L·d) ↓	37% ↑	[49]
1 mmol/L TMEDA	15%	*Chlorella* sp. *L166*	0.072 g/(L·d) ↑	43.29% ↑	[50]
CA–GA beads	Air	*Nannochloropsis salina*	0.040 g/(L·d) ↑	No data	[13]
Immobilized CA on ElectrospunNanofibers	15%	*Dunaliella. tertiolecta ATCC 30929*	6.8 × 10^5^ cells/(mL·d) ↑	No data	[64]
Metal–organic frameworks	1.50%	*Scenedesmus obliquus*	0.240 g/(L·d) ↑	21.6% ↑	[65]
CA encapsulation using bamboo cellulose scaffolds	5%	*Chlorella vulgaris*	0.275 g/(L·d) ↑	No data	[67]

↑ indicates that the indicators of the experimental group have improved compared with the control group, ↓ indicates that the experimental group’s indicators have improved compared with the control group.

## Data Availability

Not applicable.
